# A deep learning-based method for predicting the frequency classes of drug side effects based on multi-source similarity fusion

**DOI:** 10.1093/bioinformatics/btaf319

**Published:** 2025-05-27

**Authors:** Haochen Zhao, Dingxi Li, Jian Zhong, Xiao Liang, Guihua Duan, Jianxin Wang

**Affiliations:** Hunan Provincial Key Lab on Bioinformatics, School of Computer Science and Engineering, Central South University, Changsha 410083, China; Hunan Provincial Key Lab on Bioinformatics, School of Computer Science and Engineering, Central South University, Changsha 410083, China; Hunan Provincial Key Lab on Bioinformatics, School of Computer Science and Engineering, Central South University, Changsha 410083, China; Hunan Provincial Key Lab on Bioinformatics, School of Computer Science and Engineering, Central South University, Changsha 410083, China; Hunan Provincial Key Lab on Bioinformatics, School of Computer Science and Engineering, Central South University, Changsha 410083, China; Hunan Provincial Key Lab on Bioinformatics, School of Computer Science and Engineering, Central South University, Changsha 410083, China

## Abstract

**Motivation:**

Drug side effects refer to harmful or adverse reactions that occur during drug use, unrelated to the therapeutic purpose. A core issue in drug side effect prediction is determining the frequency of these drug side effects in the population, which can guide patient medication use and drug development. Many computational methods have been developed to predict the frequency of drug side effects as an alternative to clinical trials. However, existing methods typically build regression models on five frequency classes of drug side effects and tend to overfit the training set, leading to boundary handling issues and the risk of overfitting.

**Results:**

To address this problem, we develop a multi-source similarity fusion-based model, named multi-source similarity fusion (MSSF), for predicting five frequency classes of drug side effects. Compared to existing methods, our model utilizes the multi-source feature fusion module and the self-attention mechanism to explore the relationships between drugs and side effects deeply and employs Bayesian variational inference to more accurately predict the frequency classes of drug side effects. The experimental results indicate that MSSF consistently achieves superior performance compared to existing models across multiple evaluation settings, including cross-validation, cold-start experiments, and independent testing. The visual analysis and case studies further demonstrate MSSF’s reliable feature extraction capability and promise in predicting the frequency classes of drug side effects.

**Availability and implementation:**

The source code of MSSF is available on GitHub (https://github.com/dingxlcse/MSSF.git) and archived on Zenodo (DOI: 10.5281/zenodo.15462041).

## 1 Introduction

Drugs are a series of small molecular compounds that are absorbed by the human body through methods such as injection or ingestion and interact with relevant targets within the body. Generally, their effects on the human body should be expected and consistent with the described efficacy. However, due to the complex chemical nature of drugs and individual differences among users, some drugs may produce many unexpected side effects, known as drug side effects (Evans and McLeod 2003, Oun *et al.* 2018). A core issue in the study of drug side effects is the prediction of their frequency. Accurately estimating the frequency of drug side effects can effectively guide clinical patient medication and reduce the risk for pharmaceutical companies in drug development, as the occurrence of side effects can lead to the withdrawal of drugs from the market, resulting in significant economic losses in research and development (Classen *et al.* 1997, Ahmed *et al.* 2002). However, traditional clinical trials to study drug side effects are time-consuming and expensive. In contrast, using computational methods to identify drug side effect frequencies early is more cost-effective and offers substantial practical benefits (Guo *et al.* 2025, Zhao *et al.* 2025).

In the field of drug side effect identification, early studies typically focused on predicting the associations between drugs and side effects, that is, whether a relationship exists between a drug and a side effect (Zhao *et al.* 2018, 2019, Ding *et al.* 2019a, 2019b, Liang *et al.* 2020). However, these methods make only a limited contribution to the benefit-risk assessment of drugs. Recently, researchers have shifted their attention to the prediction of frequencies of drug side effects. Accurate estimation of the frequencies of drug side effects is critical to patient care in clinical practice and pharmaceutical companies because it reduces the risk of withdrawing drugs from the market. Moreover, the precise prediction of drug side effect frequencies is crucial for identifying high-risk drug–drug interactions and optimizing combination therapy regimens (Masumshah *et al.* 2021, Masumshah and Eslahchi 2023). Prior studies have demonstrated that drugs associated with high-frequency, high-risk drug side effects occupy a central role in clinically significant drug-drug interactions. In 2020, Galeano *et al.* (2020) used the SIDER database and statistical methods to transform various types of drug side effect frequency records into five frequency classes: very rare, rare, infrequent, frequent, and very frequent. They also constructed the first benchmark dataset to predict drug side effect frequencies. Based on this dataset, researchers have proposed numerous methods for predicting the frequencies of drug side effects. For example, Galeano *et al.* (2020) proposed a non-negative matrix factorization method to provide interpretable predictions for the frequencies of drug side effects. Zhao *et al.* (2021) built a drug-side effect bipartite graph and used an attention mechanism to learn feature representations from the neighbors of drug nodes and side effect nodes to predict the corresponding drug side effect scores. Xu *et al.* (2022) proposed a graph attention method using drug molecular structures and side effect semantic information to predict drug side effect frequencies. Zhao *et al.* (2022) utilized various similarities between drugs and side effects through convolutional neural networks and multilayer perceptrons to predict unknown drug side effect frequencies. In recent work, Park *et al.* (2025) employed direct drug-protein target information to represent drug carriers and used the Adaboost technique to predict the side effect frequencies of new drugs. Liu *et al.* (2024) applied a multimodal fusion strategy, incorporating multimodal information (including biomedical texts, molecular structures, and attribute similarities) to predict unknown drug side effect frequencies.

Although existing approaches have shown promising results, they typically formulate frequency prediction of drug side effects as a regression task. However, the benchmark dataset uses discrete integer labels (ranging from 0 to 4), not continuous values. This mismatch may lead to ambiguity when mapping continuous outputs to discrete categories, particularly in cases where class boundaries are unclear or overlapping. Moreover, most deep learning-based models use conventional architectures (e.g. shallow neural networks or basic graph-based models) and lack mechanisms to dynamically weigh the importance of different features or entity interactions. These limitations hinder their ability to model nuanced patterns, especially in rare or new drug scenarios.

To address these problems, we propose a multi-class model, named multi-source similarity fusion (MSSF), for predicting drug side effect frequency classes based on multi-source similarity fusion. Specifically, we collect multi-modal data on drugs and side effects from STITCH (Kuhn *et al.* 2008), DrugBank (Wishart *et al.* 2018), and ADReCS (Cai *et al.* 2015), and calculate 11 similarity matrices for drugs and 4 similarity matrices for side effects based on these multi-modal data. Then, we generate multiple feature vectors for each drug and side effect by accessing the rows corresponding to these matrices. For the feature vectors of drugs and side effects, we apply three combination operations to generate three combined vectors, which are then fed into three separate feature extraction modules to obtain the latent representations of drug–side effect pairs. These modules include two autoencoders (EN-con and EN-add) and a convolutional neural network (CNN). Next, the latent features are fused using the attention mechanism, which captures the multi-faceted, in-depth relationships between drugs and side effects. We then construct a deep Bayesian variational inference (BVI) module to capture the data distribution features, enhancing the model’s ability to fit the underlying data patterns more accurately and reducing the risk of overfitting. By defining the frequency prediction of drug side effects as a multi-classification problem, our model aligns better with the dataset structure, leading to improved predictive performance. Experimental results show that our model outperforms previous methods in ten-fold cross-validation and cold start experiments. Ablation studies verify the effectiveness of each module, confirming their contribution to the overall performance. Furthermore, visualization experiments and case studies demonstrate that our model possesses reliable feature extraction capabilities and can accurately predict the frequency classes of side effects for new drugs, underscoring its robustness and practicality in real-world applications.

## 2 Materials and methods

### 2.1 Datasets

The benchmark dataset for drug side effect frequency is derived from Galeano *et al.* (2020), which contains 757 drugs and 994 side effects. There are a total of 37,387 known frequency entries, and all frequency values are divided into five corresponding classes: very rare (frequency class = 1), rare (frequency class = 2), infrequent (frequency class = 3), frequent (frequency class = 4), and very frequent (frequency class = 5). Details on the number of samples and their proportions for each frequency class are given in Supplementary Fig. S1, available as supplementary data at *Bioinformatics* online. For convenience, let *n* (=757) and *m* (=994) be the number of different drugs and side effects in the benchmark dataset, respectively. Then, we can construct an n×m matrix *DSA* to represent the frequency classes between drugs and side effects in the benchmark dataset. Suppose the frequency class between a drug and a side effect is known in the benchmark dataset, the element’s value at the corresponding position of *DSA* is set to the corresponding frequency class.

Moreover, we introduce Zhao’s dataset (Zhao *et al.* 2018), constructed based on the SIDER database (Kuhn *et al.* 2010), for an independent test. Specifically, we identify overlapping drugs between the benchmark dataset and Zhao’s dataset and then collect their side effect frequency classes from the benchmark dataset to construct the training dataset. This training dataset includes 31,452 drug-side effect frequency pairs for 588 drugs. The remaining part of the benchmark dataset, consisting of 5935 drug-side effect frequency pairs for 169 drugs, is used as the independent test set.

To calculate the features of drugs and side effects, we collect relevant data on drugs and side effects from multiple databases, such as STITCH, DrugBank, and ADReCS. We then calculate 11 drug similarity matrices, including $SMD_{\mathrm{Similarity}}$, $SMD_{\mathrm{Experimental}}$, $SMD_{\mathrm{Database}}$, $SMD_{\mathrm{Text}}$, $SMD_{\mathrm{Combined}}$, $SMD_{\mathrm{Structure}}$, $SMD_{\mathrm{Word}}$, $SMD_{\mathrm{Target}}$, $SMD_{\mathrm{Pathway}-\mathrm{enzyme}}$, $SMD_{\mathrm{DIPF}}$, and $SMD_{\mathrm{DIPA}}$ and 4 side effect similarity matrices, including $SME_{\mathrm{Semantic}}$, $SME_{\mathrm{Word}}$, $SME_{\mathrm{DIPA}}$, and $SME_{\mathrm{DIPF}}$, according to the similarity calculation methods of Zhao *et al.* (2022). Each drug matrix is a 757×757 square matrix, and each side effect matrix is a 994×994 square matrix. Specifically, STITCH describes the associations between chemicals from five aspects: “similarity,” “experimental,” “database,” “text mining,” and “combined score.” By collecting the five types of association scores between drugs in the benchmark dataset, we can construct $SMD_{\mathrm{Similarity}}$, $SMD_{\mathrm{Experimental}}$, $SMD_{\mathrm{Database}}$, $SMD_{\mathrm{Text}}$, and $SMD_{\mathrm{Combined}}$. Since all chemical-chemical association scores in STITCH range from 1 to 1000, all scores are divided by 1000 to ensure the drug similarity values range from 0 to 1. The self-similarity for each drug is set to 1. $SMD_{\mathrm{Structure}}$ represents the structural similarity matrix between drugs, calculated based on drug Morgan fingerprint vectors generated using the RDKit (Lovrić *et al.* 2019) toolkit and computed according to the Jaccard score. $SMD_{\mathrm{Target}}$ is calculated based on 847 protein targets stored in the DrugBank database associated with the drugs in the benchmark dataset. We first construct an 847-dimensional vector for each drug, indicating its associations with the protein targets, and then compute the cosine similarity between these vectors to obtain the similarity matrix $SMD_{\mathrm{Target}}$. We also obtain 100-dimensional drug substructure vectors using the unsupervised machine learning method Mol2vec (Jaeger *et al.* 2018). By calculating the cosine similarity coefficients between the substructure vectors, we obtain $SMD_{\mathrm{Word}}$. $SMD_{\mathrm{DIPF}}$ and $SMD_{\mathrm{DIPA}}$ are calculated based on *DSA*. $SMD_{\mathrm{DIPF}}$ is computed by calculating the cosine similarity between the row vectors corresponding to the drugs in *DSA*. $SMD_{\mathrm{DIPA}}$ is obtained by converting the row vectors corresponding to the drugs in *DSA* into 0/1 vectors (where a record exists for a drug and a side effect in the benchmark dataset, the corresponding position is set to 1, otherwise 0), and then calculating the cosine similarity between these vectors. $SMD_{\mathrm{Pathway}-\mathrm{enzyme}}$ is calculated based on metabolic pathway and enzyme interaction data obtained from DrugBank. Specifically, we first collect 309 pathway records associated with the 757 drugs in the benchmark dataset to construct a drug–pathway association matrix, where an entry is set to 1 if a drug is involved in a given pathway. Next, we extract pathway–enzyme associations from DrugBank, involving 592 enzymes, and construct a corresponding pathway–enzyme matrix. By multiplying the drug–pathway matrix with the pathway–enzyme matrix, we obtain a drug–pathway–enzyme matrix. Cosine similarity between its row vectors yields the $SMD_{\mathrm{Pathway}-\mathrm{enzyme}}$.

For side effects, there are a total of four similarity matrices. $SME_{\mathrm{Semantic}}$ is the semantic similarity matrix calculated based on the directed acyclic graphs of side effects. A directed acyclic graph is constructed using all semantic descriptors related to the side effect. By calculating the similarity between nodes in the directed acyclic graphs corresponding to different side effects, the semantic similarity between side effects can be generated. $SME_{\mathrm{Word}}$ is the word vector similarity matrix obtained by calculating the cosine similarity of the pre-trained 300-dimensional word vectors of side effects. The calculation processes of $SME_{\mathrm{DIPA}}$ and $SME_{\mathrm{DIPF}}$ are similar to $SMD_{\mathrm{DIPF}}$ and $SMD_{\mathrm{DIPA}}$ of drugs. $SME_{\mathrm{DIPF}}$ is computed by calculating the cosine similarity between the column vectors corresponding to the side effects in *DSA*. $SME_{\mathrm{DIPA}}$ is obtained by converting the column vectors corresponding to the side effects in *DSA* into 0/1 vectors and then calculating the cosine similarity between these vectors.

### 2.2 Multi-source similarity fusion

**Figure 1.**
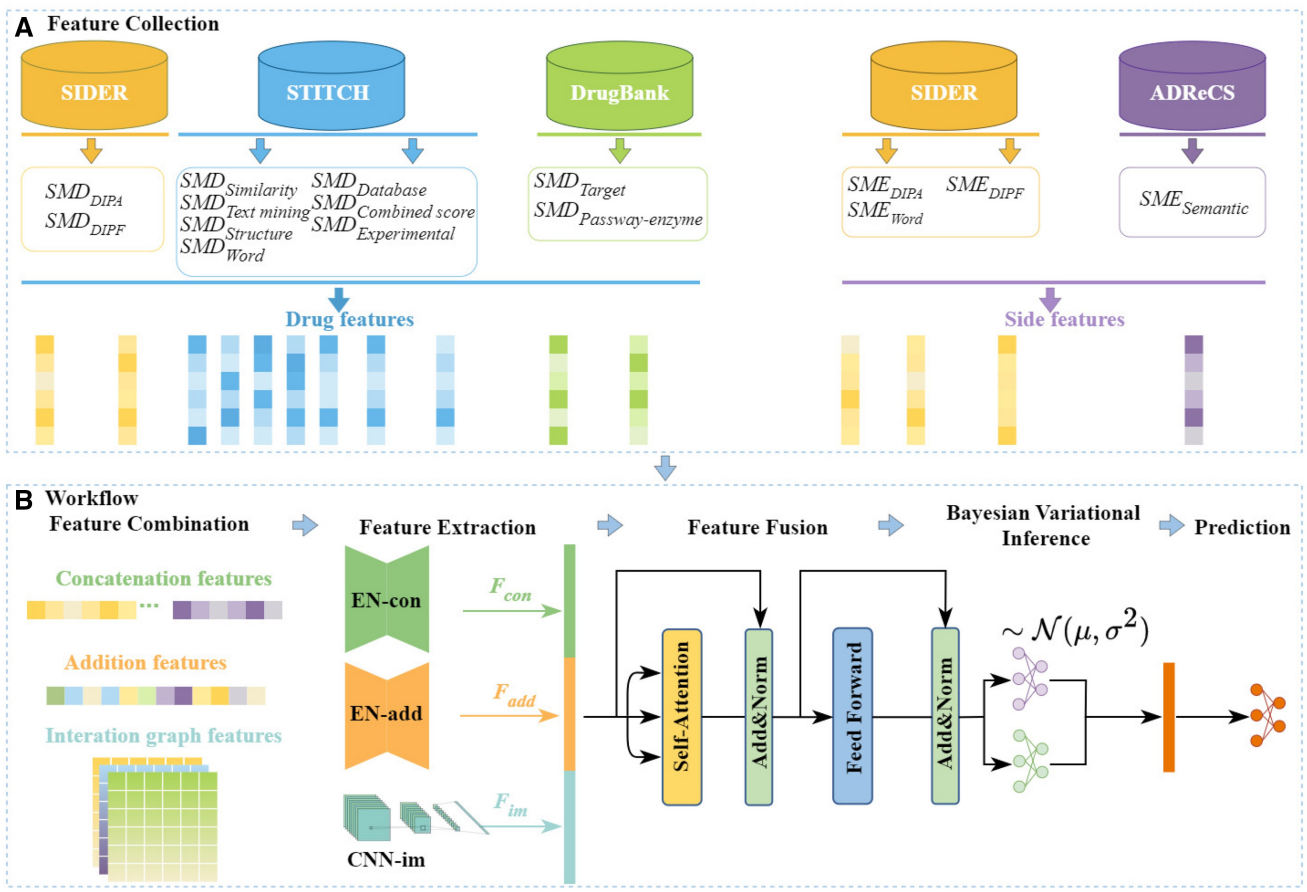
The overview of MSSF. (A) Feature collection process; (B) The overall workflow of the model, including feature combination, feature extraction, feature fusion, Bayesian variational inference, and final classification.

In this article, we propose the MSSF for predicting the frequency classes of drug side effects based on multi-source similarity fusion. As shown in Fig. 1, the model can be described through the following five steps:(i) Feature collection: Collecting drug and side effect feature vectors based on various types of similarity matrices; (ii) Latent representation extraction: Preprocessing the feature vectors of drugs and side effects and then forming 3 feature combinations of drug-side effect pairs, which include feature concatenation, feature addition, and feature interaction map. These are input separately into EN-con, EN-add, and CNN-im modules for latent representation extraction; (iii) Latent representation fusion: Fusing the latent representations using an attention mechanism to form the fused representations; (iv) Bayesian variational inference: Inputting the fused representations into a Bayesian variational inference module to obtain the final representations for drug-side effect pairs and (v) Prediction: Inputting the representations into a multi-layer perceptron (MLP) to predict the corresponding frequency classes between drugs and side effects. Next, we take the drug-side effect pair $d_{i}$-$s_{j}$ as an example and illustrate the workflow of our model.

#### 2.2.1 Feature collection

We firstly define the similarity matrix sets for drugs and side effects, respectively, as $S_{\mathrm{Drug}}=\{SMD_{\mathrm{Similarity}}$, $SMD_{\mathrm{Experimental}}$, $SMD_{\mathrm{Database}}$, $SMD_{\mathrm{Text}}$, $SMD_{\mathrm{Combined}}$, $SMD_{\mathrm{Structure}}$, $SMD_{\mathrm{Target}}$, $SMD_{\mathrm{Word}}$, $SMD_{\mathrm{Pathway}-\mathrm{enzyme}}$, $SMD_{\mathrm{DIPA}}$, $SMD_{\mathrm{DIPF}}\}$ and $S_{\mathrm{Side}-\mathrm{effect}}=\{SME_{\mathrm{Semantic}}$, $SME_{\mathrm{Word}}$, $SME_{\mathrm{DIPA}}$, $SME_{\mathrm{DIPF}}\}$. Then, we can collect the corresponding row vectors in the above matrices to construct the feature vectors of drugs or side effects. For $d_{i}$, the *p*-th feature vector $F_{d_{i}}^{p}=S_{\mathrm{Drug}}(p)[i]$, where $S_{\mathrm{Drug}}(p)[i]$ represents the *i*-th row of the *p*-th element in set $S_{\mathrm{Drug}}$ (with *p* ranging from 1 to 11). Similarly, for $s_{j}$, the *q*-th feature vector $F_{s_{j}}^{q}=S_{\mathrm{Side}-\mathrm{effect}}(q)[j]$, where $S_{\mathrm{Side}-\mathrm{effect}}(q)[j]$ represents the *j*-th row of the *q*-th element in set $S_{\mathrm{Side}-\mathrm{effect}}$ (with *q* ranging from 1 to 4). Each drug feature vector has dimensions of 757 and each side effect feature vector has dimensions of 994. Thus, we can obtain 11 feature vectors for $d_{i}$ and 4 feature vectors for $s_{j}$, as illustrated in Fig. 1A.

#### 2.2.2 Latent feature extraction

Based on the 11 feature vectors of $d_{i}$ and the 4 feature vectors of $s_{j}$, we obtain three types of combined features for $d_{i}$-$s_{j}$: concatenation feature, addition feature, and interaction graph feature, as illustrated in Fig. 1B. These feature combinations are then input into three feature extraction modules, namely EN-con, EN-add, and CNN-im, respectively, for latent representation extraction. Below is a detailed explanation of the preprocessing steps and implementation details for each module:

##### 2.2.2.1 Auto-encoder

The concatenation feature of $d_{i}$-$s_{j}$ is obtained by merging all the features of $d_{i}$ and $s_{j}$, resulting in a dimension of (11×m+4×n). To construct the addition feature for $d_{i}-s_{j}$, we first perform element-wise summation across the 11 drug feature vectors to obtain an *m*-dimensional vector, and across the four side effect vectors to obtain an *n*-dimensional vector. These two vectors are then concatenated to form a feature vector of dimension (m+n). These two feature vectors are then fed into the EN-con and EN-add modules, respectively, which are both auto-encoders with similar structures. Auto-encoders are widely used in deep learning (Deng *et al.* 2022) due to their ability to generate meaningful representations that are highly beneficial for identifying the latent features necessary for various tasks (Bank *et al.* 2023). An auto-encoder is composed of an encoder and a decoder. The encoder primarily consists of two linear layers, with a self-attention layer inserted between them. This configuration enables the auto-encoder to focus on the most relevant features. The decoder is composed of two linear layers. The latent feature vectors, $F_{\mathrm{con}}$ and $F_{\mathrm{add}}$, are derived from the encoder outputs, with $F_{\mathrm{con}}$ obtained from EN-con and $F_{\mathrm{add}}$ from EN-add. The outputs of the decoders are subsequently used to compute auxiliary losses. The self-attention layer in the encoder of an auto-encoder is as follows:


(1) $$\mathrm{Attention}=\mathrm{Softmax}(\frac{(X_{\mathrm{Att}}W_{Q})\times {\left(X_{\mathrm{Att}}W_{K}\right)}^{T}}{\sqrt{d_{k}}})X_{Att}W_{V}$$

where $X_{\mathrm{Att}}$ is the matrix formed by stacking the output vectors of the first linear layer in the encoder, and $W_{Q}$, $W_{K}$, and $W_{V}$ are the parameter matrices used to compute the query, key, and value, respectively. The output $F_{e}$ is obtained through a residual connection:


(2) $$F_{e}=\mathrm{ReLU}(LN(\mathrm{Attention}+X_{\mathrm{Att}}))+X_{\mathrm{Att}}$$

where *LN* represents Layer Normalization, which is commonly used in transformer architectures to accelerate convergence, and ReLU is the activation function. The use of residual connections helps prevent gradient vanishing and information loss, thereby enhancing the learning capability of the network (He *et al.* 2016).

##### 2.2.2.2 Drug-side effect interaction map

We extract interaction relationships by performing an outer product on different drug and side effect features. The resulting two-dimensional features are then processed using the CNN-im module to extract latent features. Specifically, we first use a linear layer to extract the potential representations from all the features of $d_{i}$ and $s_{j}$. Then, we compute the cross product of each representation of $d_{i}$ with each representation of $s_{j}$, resulting in 44 (11×4) feature interaction maps:


(3) $$\mathrm{CrossD}S_{k}^{i,j}=X_{d_{i}}^{p}⊗X_{s_{j}}^{q}$$

where $\mathrm{CrossD}S_{k}^{i,j}$ represents the *k*-th interaction map, which is generated by the outer product of the *p*-th representation of $d_{i}$ ($X_{d_{i}}^{p}$) and the *q*-th representation of $s_{j}$ ($X_{s_{j}}^{q}$). We then employ the CNN-im module, consisting of three layers, each made up of a 2D convolutional layer, batch normalization, and an activation function, to extract representations from the interaction maps. By flattening the output of CNN-im, we obtain the latent feature $F_{im}$.

#### 2.2.3 Latent feature fusion

In the latent representation fusion module, we first concatenate $F_{\mathrm{con}}$, $F_{\mathrm{add}}$, and $F_{im}$ to form a concatenation vector:


(4) $$X_{f}=\mathrm{Concat}(F_{\mathrm{con}},F_{\mathrm{add}},F_{im})$$

where *Concat* denotes the concatenation operation. The concatenated vector is then fed into a self-attention layer with the same architecture as the EN-con and EN-add modules, to generate the fused feature representation $F_{f}$.

#### 2.2.4 Bayesian variational inference

To reduce the risk of overfitting and improve generalization, we introduce a BVI module into our model. This choice is motivated by the specific challenges of our task: drug side effect frequencies are often inferred from limited, imbalanced, or noisy clinical data, making uncertainty quantification essential for reliable prediction. Unlike deterministic models that yield fixed-point estimates, BVI provides a probabilistic framework for learning latent representations by approximating the posterior distribution of the model parameters. This allows the model not only to make predictions but also to estimate the uncertainty associated with those predictions. In high-stakes biomedical applications such as drug safety, this uncertainty awareness is crucial for downstream decision-making and interoperability. Moreover, BVI incorporates prior distributions and performs posterior regularization, which helps mitigate overfitting, especially when training data is sparse or skewed across frequency classes. BVI module consists of two linear layers: one to estimate the mean $\mu (F_{f})$ of the fused features, and another to estimate the log-variance $\text{log}\;\sigma^{2}(F_{f})$. The latent feature vector $F_{l}$ is then sampled using the reparameterization trick to allow gradient-based optimization:


(5) $$F_{l}=\mu (F_{f})+\epsilon \cdot \sigma (F_{f})$$

where ϵ∼N(0,I) and $\sigma (F_{f})=\text{exp}(0.5\cdot \;\text{log}\;\sigma^{2}(F_{f}))$.

### 2.3 Model training

In the prediction module, we use an MLP as the classifier to predict the frequency classes of drug side effects, which consist of two fully connected hidden layers. The objective of MSSF is to accurately classify drug side effect frequencies into five distinct classes. Consequently, the primary loss function for the model is the classification loss, calculated using the Binary Cross-Entropy (BCE) function ($L_{\mathrm{BCE}}$). Additionally, in the latent feature extraction part of the model, there are two auto-encoder modules. We employ mean squared error (MSE) to compute the losses for the auto-encoders ($L_{MSE_{1}}$ and $L_{MSE_{2}}$), which serve as auxiliary losses to enhance the model’s performance. Moreover, we compute the KL divergence based on the Bayesian variational inference module as a regularization term to prevent overfitting and improve the model’s generalization ability:


(6) $$\begin{array}{l} L_{\mathrm{BCE}}=\mathrm{BCE}(y_{\mathrm{true}},y_{\mathrm{pred}})\\ L_{MSE_{1}}=\alpha_{1}\cdot \mathrm{MSE}(x_{\mathrm{tru}e_{1}},x_{\mathrm{pre}d_{1}})\\ L_{MSE_{2}}=\alpha_{2}\cdot \mathrm{MSE}(x_{\mathrm{tru}e_{2}},x_{\mathrm{pre}d_{2}})\\ KL(\mu ,\sigma )=\frac{1}{2}\sum_{i=1}^{d}(1+2ln(\sigma^{i})-{\left(\mu^{i}\right)}^{2}-{\left(\sigma^{i}\right)}^{2})\\ L_{\mathrm{total}}=L_{BCE}+L_{MSE_{1}}+L_{MSE_{2}}+\alpha_{3}KL(\mu ,\sigma )+\gamma R(\theta ) \end{array}$$

where $y_{\mathrm{true}}$ and $y_{\mathrm{pred}}$ represent the corresponding labels and the model’s prediction scores, respectively. $x_{\mathrm{tru}e_{1}}$ and $x_{\mathrm{pre}d_{1}}$ represent the output of the EN-con encoder and the output of the EN-con decoder, respectively. $x_{\mathrm{tru}e_{2}}$ and $x_{\mathrm{pre}d_{2}}$ represent the output of the EN-add encoder and the output of the EN-add decoder, respectively. *d* is the dimension of $F_{l}$, γR(θ) is the L2 regularization term, with γ as its weight, θ as the model parameters, and $\alpha_{1},\alpha_{2},\alpha_{3}$ are three small real numbers that serve as weights. There are some important hyperparameters in our model, including learning rate, batch size, dropout rate, *d*, and γ. We determine the hyperparameters using grid search on the benchmark dataset and the independent set. In the grid search, the learning rate is in the range of [1e-3, 5e-4, 1e-4, 5e-5, and 1e-5], dropout rate in [0.1, 0.2, 0.3, 0.4, 0.5, 0.6, 0.7, 0.8, and 0.9], batch size in [32, 64, 128, and 256], *d* in [16, 32, 64, 128, and 256] and γ in [1e-1, 1e-2, 1e-3, 1e-4, and 1e-5]. For the benchmark dataset, the optimal values are determined to be 1e-4 for the learning rate, 0.4 for the dropout rate, 128 for the batch size, 64 for *d*, and 1e-5 for γ (see Supplementary Tables S1–S5, available as supplementary data at *Bioinformatics* online). For the independent set, the optimal values are determined to be 5e-5 for the learning rate, 0.2 for the dropout rate, 64 for the batch size, 32 for *d*, and 1e-5 for γ (see Supplementary Tables S6–S10, available as supplementary data at *Bioinformatics* online).

## 3 Results

### 3.1 Comparison with other methods

We employ ten-fold cross-validation on the benchmark dataset to compare our method with the baselines. In the ten-fold cross-validation, the drug-side effect pairs with known frequency classes are randomly divided into 10 parts. In the *k*-th fold, the *k*-th part of the data is used as the test set, while the remaining nine parts serve as the training set. To evaluate the performance of MSSF, we compare it with the methods proposed by Galeano *et al.* (2020), MGPred (Zhao *et al.* 2021), SDPred (Zhao *et al.* 2022), DSGAT (Xu *et al.* 2022), Park (Park *et al.* 2025), and HMMF (Liu *et al.* 2024). Since the previously proposed methods predict the frequency of drug side effects as regression values rather than classes, we use kernel density estimation to derive the probability density function (PDF) for each frequency class. Then, using the PDF and maximum likelihood estimation, we define classification decision thresholds as described in Galeano *et al.* (2020), which allows us to calculate the relevant metrics. The specific calculation methods are detailed in Supplementary Section S3, available as supplementary data at *Bioinformatics* online. To evaluate the performance of the methods, we chose to use eight metrics for performance evaluation: Accuracy (ACC), Weighted F1 score (Weighted F1), Macro F1 score (Macro F1), Cohen’s Kappa Score (κ), Matthews Correlation Coefficient (MCC), Macro precision (Precision), Macro recall (Recall), and Macro-Averaged Area Under the Precision-Recall Curve (AUPR) (The detail of the calculation process see Section S4, available as supplementary data at *Bioinformatics* online). For all baseline methods, we perform hyperparameter tuning using ten-fold cross-validation on the benchmark dataset and the independent test to ensure a fair comparison. The results of the comparative experiments are shown in Table 1. Compared with the second-best method, HMMF, our method achieves improvements of 15.9%, 14.2%, 12.5%, 23.6%, 22.6%, 19.6%, 3.8%, and 15.7% in ACC, Weighted F1, Macro F1, Kappa, MCC, Precision, Recall, and AUPR, respectively. Meanwhile, we conduct paired t-tests between the results of each fold in the ten-fold cross-validation of MSSF and the corresponding results of other models. The relevant *p*-values are shown in Table 2. The *P*-values indicate that the performance improvements of our proposed model over the baseline methods are statistically significant in most cases (*P* < 0.05). These results demonstrate that feature extraction from multiple perspectives, fusion strategies, and Bayesian variational inference significantly enhance the classification performance of drug side effects across different classes.

**Table 1.** Performance comparison of different methods on the benchmark dataset using ten-fold cross-validation.

Model name	ACC	Weighted F1	Macro F1	Kappa	MCC	Precision	Recall	AUPR
Geleano’s model	0.3869	0.4023	0.3636	0.2134	0.2277	0.3686	0.4664	0.3604
MGPred	0.5187	0.5260	0.4952	0.3409	0.3503	0.4914	0.5333	0.5041
DSGAT	0.4555	0.4699	0.4207	0.2773	0.2889	0.4100	0.5024	0.4121
SDPred	0.5868	0.5937	0.5576	0.4243	0.4314	0.5527	0.5888	0.5749
Park’s model	0.3771	0.3868	0.3545	0.1902	0.2018	0.3512	0.4367	0.3506
HMMF	0.6254	0.6314	0.6053	0.4732	0.4784	0.5893	0.6396	0.6259
MSSF	**0.7246**	**0.7212**	**0.6809**	**0.5847**	**0.5863**	**0.7046**	**0.6641**	**0.7272**

The best-performing result in each column is highlighted in bold.

**Table 2.** The *P*-values of paired *t*-tests comparing MSSF with other models.

	Gelleano’s model	MGPred	DSGAT	SDPred	Park’s model	HMMF
ACC	$6.57\times 10^{-23}$	$2.31\times 10^{-12}$	$1.36\times 10^{-23}$	$1.24\times 10^{-14}$	$3.33\times 10^{-18}$	$1.84\times 10^{-13}$
Weight F1	$5.48\times 10^{-20}$	$2.40\times 10^{-12}$	$3.08\times 10^{-23}$	$1.33\times 10^{-14}$	$7.54\times 10^{-18}$	$2.14\times 10^{-13}$
Macro F1	$1.55\times 10^{-23}$	$8.20\times 10^{-14}$	$6.06\times 10^{-21}$	$1.20\times 10^{-13}$	$1.08\times 10^{-19}$	$1.15\times 10^{-10}$
kappa	$2.02\times 10^{-22}$	$2.59\times 10^{-14}$	$2.25\times 10^{-21}$	$1.80\times 10^{-15}$	$7.09\times 10^{-23}$	$8.40\times 10^{-13}$
MCC	$1.91\times 10^{-22}$	$1.90\times 10^{-14}$	$1.08\times 10^{-21}$	$1.81\times 10^{-15}$	$2.14\times 10^{-22}$	$6.89\times 10^{-13}$
Precision	$1.70\times 10^{-22}$	$3.31\times 10^{-18}$	$2.36\times 10^{-21}$	$1.29\times 10^{-15}$	$6.84\times 10^{-23}$	$1.61\times 10^{-12}$
Recall	$5.81\times 10^{-15}$	$9.64\times 10^{-12}$	$6.69\times 10^{-14}$	$5.77\times 10^{-9}$	$2.02\times 10^{-17}$	$4.21\times 10^{-3}$
AUPR	$2.14\times 10^{-26}$	$2.20\times 10^{-13}$	$2.04\times 10^{-24}$	$2.94\times 10^{-13}$	$2.07\times 10^{-21}$	$1.43\times 10^{-8}$

Moreover, to evaluate MSSF’s ability to predict the frequency classes of side effects for new drugs, we design a cold start experiment and an independent test. In the cold start experiment, we divide all drugs into ten parts and conduct a ten-fold cross-validation experiment. In the *k*-th fold, the drug-side effect pairs related to the drugs in the *k*-th part are used as the test set, while the remaining nine parts are used as the training set. For the independent test, we use the drug-side effect pairs related to the overlapping drugs between the benchmark dataset and Zhao’s dataset (Zhao *et al.* 2018) as the training set, with the remaining part of the benchmark dataset serving as the test set. Since the methods by Galeano *et al.* and MGPred rely on known frequencies of drug side effects and cannot predict side effect frequencies for new drugs, we do not evaluate these two methods. Additionally, because side effects of new drugs cannot be obtained, we remove the two drug similarity matrices ($SMD_{\mathrm{DIPA}},SMD_{\mathrm{DIPF}}$) and the two side effect similarity matrices ($SME_{\mathrm{DIPA}},SME_{\mathrm{DIPF}}$) in the cold start experiment and the independent test. The results of our model and other methods in the cold start experiments and independent tests are shown in Tables 3 and 4, respectively. These results show that our model also outperforms others in predicting the frequency classes of side effects for new drugs under both cold start and independent test conditions.

**Table 3.** Performance comparison of different methods in the cold start condition.

Model name	ACC	Weighted F1	Macro F1	Kappa	MCC	Precision	Recall	AUPR
DSGAT	0.3562	0.3642	0.2930	0.1506	0.1603	0.3025	0.3443	0.3060
SDPred	0.4286	0.4330	0.3726	0.2276	0.2374	0.3942	**0.4137**	0.3825
Park’s model	0.3347	0.3546	0.2999	0.1408	0.1495	0.3085	0.3661	0.3063
HMMF	0.4282	0.4384	0.3692	0.2155	0.2206	0.3629	0.4047	0.3510
MSSF	**0.5601**	**0.5256**	**0.3896**	**0.2745**	**0.2900**	**0.4686**	0.3780	**0.4073**

The best-performing result in each column is highlighted in bold.

**Table 4.** Performance comparison of different methods in the independent test conditions.

Model name	ACC	Weighted F1	Macro F1	Kappa	MCC	Precision	Recall	AUPR
DSGAT	0.1948	0.1403	0.1574	0.0683	0.0926	0.2401	0.3130	0.2799
SDPred	0.3842	0.3938	0.3400	0.1639	0.1741	0.3372	**0.4229**	0.3565
Park’s model	0.3857	0.4017	0.3028	0.1694	0.1799	0.3192	0.3760	0.3169
HMMF	0.4162	0.4284	0.3236	0.1717	0.1785	0.3364	0.3832	0.3407
MSSF	**0.5606**	**0.5282**	**0.3940**	**0.2425**	**0.2549**	**0.4704**	0.3681	**0.3897**

The best-performing result in each column is highlighted in bold.

### 3.2 Ablation study

To study the impact of important modules on the complete model, we conducted a ten-fold cross-validation experiment based on the benchmark dataset, gradually removing each component of the latent representation extraction module and the Bayesian variational inference module to evaluate their importance. The variants “only EN-con,” “only EN-add,” and “only CNN-im” represent the use of only EN-con, EN-add, and CNN-im in the feature extraction module, respectively. The variants “w/o EN-con,” “w/o EN-add,” and “w/o CNN-im” represent the removal of EN-con, EN-add, and CNN-im from the feature extraction module, respectively. The variant “w/o Bayes” represents removing the Bayesian variational inference module before the final classification. The results indicate that each proposed module contributes to the overall model’s prediction performance (see Table 5).

**Table 5.** Performance comparison of different model variants in the ablation study.

Model name	ACC	Weighted F1	Macro F1	Kappa	MCC	Precision	Recall	AUPR
only EN-con	0.7087	0.7054	0.6637	0.5625	0.5644	0.6799	0.6562	0.7212
only EN-add	0.7042	0.7005	0.6575	0.5553	0.5570	0.6768	0.6477	0.7147
only CNN-im	0.6780	0.6769	0.6336	0.5208	0.5226	0.6730	0.6177	0.6927
w/o EN-con	0.7111	0.7103	0.6699	0.5700	0.5703	0.6870	0.6575	0.7164
w/o EN-add	0.7156	0.7125	0.6699	0.5720	0.5731	0.6912	0.6546	0.7252
w/o CNN-im	0.7150	0.7120	0.6763	0.5722	0.5738	0.6911	0.6679	0.7239
w/o Bayes	0.7185	0.7164	0.6807	0.5799	0.5804	0.6844	**0.6806**	0.7251
MSSF	**0.7246**	**0.7212**	**0.6809**	**0.5847**	**0.5863**	**0.7046**	0.6641	**0.7272**

The best-performing result in each column is highlighted in bold.

Moreover, MSSF computes ten different drug similarities and four different side effect similarities. To assess the impact of each similarity on the overall model, we excluded one similarity feature at a time and retrained and tested our model on the benchmark dataset based on ten-fold cross-validation. The results show that each drug or side effect similarity contributes to some extent (see Tables 6 and 7). Notably, the worst result shows an accuracy of only 1.2% lower than the result using all similarity features, indicating that our model can still achieve good performance with fewer similarity features.

**Table 6.** Performance of MSSF when one drug similarity is removed.

Model name	ACC	Weighted F1	Macro F1	Kappa	MCC	Precision	Recall	AUPR
$SMD_{\mathrm{Similarity}}$	0.7180	0.7150	0.6728	0.5760	0.5770	0.6939	0.6594	0.7237
$SMD_{\mathrm{Experimental}}$	0.7167	0.7137	0.6731	0.5744	0.5757	0.6942	0.6600	0.7237
$SMD_{\mathrm{Database}}$	0.7164	0.7130	0.6706	0.5731	0.5747	0.6937	0.6566	0.7229
$SMD_{\mathrm{Text}}$	0.7175	0.7140	0.6726	0.5742	0.5759	0.6964	0.6568	0.7236
$SMD_{\mathrm{Combined}}$	0.7160	0.7127	0.6722	0.5727	0.5741	0.6919	0.6600	0.7243
$SMD_{\mathrm{Structure}}$	0.7171	0.7151	0.6745	0.5776	0.5786	0.6852	0.6699	0.7272
$SMD_{\mathrm{Target}}$	0.7173	0.7142	0.6724	0.5756	0.5772	0.6865	0.6662	0.7253
$SMD_{\mathrm{Word}}$	0.7186	0.7173	0.6773	0.5806	0.5812	0.6893	0.6702	0.7249
*SMD_DIPA_*	0.7157	0.7128	0.6708	0.5726	0.5738	0.6920	0.6569	0.7201
*SMD_DIPF_*	0.7168	0.7150	0.6756	0.5769	0.5778	0.6892	0.6678	0.7227
$SMD_{\mathrm{Passway}-\mathrm{enzyme}}$	0.7204	0.7179	0.6752	0.5801	0.5812	0.6969	0.6612	0.7230

**Table 7.** Performance of MSSF when one side similarity is removed.

Model name	ACC	Weighted F1	Macro F1	Kappa	MCC	Precision	Recall	AUPR
$SME_{\mathrm{Semantic}}$	0.7165	0.7125	0.6722	0.5721	0.5738	0.6980	0.6563	0.7233
$SME_{\mathrm{Word}}$	0.7177	0.7126	0.6690	0.5738	0.5777	0.6874	0.6615	0.7217
$SME_{DIPA}$	0.7191	0.7165	0.6770	0.5778	0.5790	0.7015	0.6601	0.7266
$SME_{DIPF}$	0.7173	0.7134	0.6713	0.5732	0.5748	0.6958	0.6558	0.7263

### 3.3 Visualization analysis

**Figure 2.**
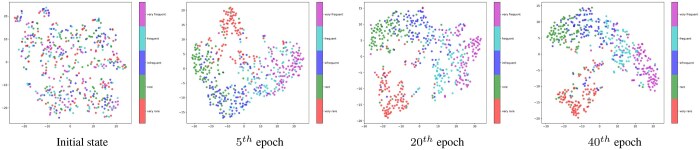
The visualization of the latent vectors on different epochs during training.

To visually demonstrate the effectiveness of our model and enhance its interpretability, we used t-SNE Marx (2024) to project the high-dimensional representation vectors of drug-side effect pairs with different frequency categories onto a two-dimensional plane for visualization. Specifically, we randomly selected 90% of the drug-side effect pairs with known frequency categories in the benchmark dataset as the training set, while the remaining 10% are used as the test set. We then recorded the latent vectors of the test set at different epochs. Next, we randomly selected 100 samples from each frequency class and applied t-SNE for two-dimensional visualization. Different colors were used to annotate the different categories. As shown in Fig. 2, the initial feature vectors from different categories are mixed together. However, as the training epochs progress, the latent vectors of different categories gradually separate. The visualization results demonstrate the learning capability of our model in predicting the frequency categories of drug side effects.

### 3.4 Case study

To evaluate the practical effectiveness of MSSF, we conduct case studies on three representative drugs. Specifically, we train the model using drug–side effect pairs with known frequency classes from the benchmark dataset and observe its predictions on the remaining unknown pairs. Specifically, for each unknown drug–side effect pair, the model outputs a five-dimensional prediction vector, where the maximum entry (max-value) indicates the predicted frequency class and, to some extent, reflects the model’s confidence in that prediction. For each drug, we collect the max-values of all its unknown side effect pairs, compute their mean, and rank the drugs by this average confidence. The top three drugs identified using this criterion are haloperidol, trimethoprim, and amoxicillin. For each of these drugs, we select the ten side effects associated with the highest max-values. We then query the OFFSIDES database (Tatonetti *et al.* 2012) to verify whether these predicted associations are supported by external evidence. Among the top 10 predictions, 8, 10, and 10 side effects for haloperidol, trimethoprim, and amoxicillin, respectively, are confirmed in the OFFSIDES database (see Supplementary Tables S11–S13, available as supplementary data at *Bioinformatics* online). These results demonstrate that MSSF is capable of not only accurately identifying the frequency classes of drug side effects but also uncovering potential side effect associations with high confidence, even in previously unannotated drug–side effect pairs.

## 4 Discussion

Accurate prediction of drug side effect frequencies is essential for evaluating the safety profiles of pharmaceuticals. In this work, we reformulate the frequency prediction task from a regression problem to a multi-class classification problem, aligning the modeling approach with the discrete nature of frequency labels in benchmark datasets. This change effectively mitigates issues related to ambiguous class boundaries and overfitting that often occur in regression-based methods.

To improve predictive performance, we introduce MSSF, a novel model that integrates diverse types of drug and side effect information through three complementary feature extraction strategies. These features are fused using a multi-head self-attention mechanism, which allows the model to capture complex cross-feature dependencies. We further incorporate Bayesian variational inference to handle uncertainty and improve robustness in classification tasks.

Our experiments demonstrate that MSSF significantly outperforms existing state-of-the-art methods across various evaluation metrics, particularly in cold-start scenarios where drug–side effect pairs are previously unseen. The ablation studies highlight the contribution of each feature extraction strategy and confirm the effectiveness of the Bayesian module. In addition, our visualization results provide insight into the training dynamics of each frequency class. Case studies further support the practical utility of our model, as it successfully predicts previously unknown side effects that are later verified by external databases.

Despite these promising results, limitations remain. The annotated frequency data for drug–side effect pairs are relatively sparse and imbalanced across classes. Moreover, our current framework primarily utilizes structured features, without incorporating richer biomedical context such as gene expression or literature-derived knowledge. Future improvements could include few-shot learning techniques to address data scarcity and the integration of additional modalities to enhance feature representations and generalization capabilities.

## 5 Conclusion

In this study, we propose a novel multi-source fusion framework (MSSF) for predicting drug side effect frequency classes. By reformulating the task as a classification problem and introducing multi-view feature extraction with self-attention and Bayesian inference, our approach achieves superior performance over existing methods. The effectiveness of the model is demonstrated through comprehensive experiments, including ablation studies, visualization, and case validations.

Our findings suggest that combining diverse drug and side effect information using advanced fusion and modeling techniques can lead to more accurate and interpretable predictions. The proposed framework holds promise for practical applications in drug safety assessment, especially in identifying high-risk side effects of novel drugs. Future work will explore the incorporation of additional data sources and improved generalization strategies to further enhance the robustness and applicability of the model.

## Supplementary Material

btaf319_Supplementary_Data

## References

[btaf319-B1] Ahmed P , Gardella J, Nanda S. Wealth effect of drug withdrawals on firms and their competitors. Financial Management 2002;31:21–41.

[btaf319-B2] Bank D , Koenigstein N, Giryes R. Autoencoders. In Machine Learning for Data Science Handbook: Data Mining and Knowledge Discovery Handbook, 353–74. Springer, 2023.

[btaf319-B3] Cai M-C , Xu Q, Pan Y-J et al Adrecs: an ontology database for aiding standardization and hierarchical classification of adverse drug reaction terms. Nucleic Acids Res 2015;43:D907–13. 25361966 10.1093/nar/gku1066 PMC4383906

[btaf319-B4] Classen DC , Pestotnik SL, Evans RS et al Adverse drug events in hospitalized patients: excess length of stay, extra costs, and attributable mortality. JAMA 1997;277:301–6. 9002492

[btaf319-B5] Deng Y , Qiu Y, Xu X et al Meta-ddie: predicting drug–drug interaction events with few-shot learning. Brief Bioinform 2022;23:bbab514. 34893793 10.1093/bib/bbab514

[btaf319-B6] Ding Y , Tang J, Guo F. Identification of drug-side effect association via semisupervised model and multiple kernel learning. IEEE J Biomed Health Inform 2019a;23:2619–32. 30507518 10.1109/JBHI.2018.2883834

[btaf319-B7] Ding Y , Tang J, Guo F. Identification of drug-side effect association via multiple information integration with centered kernel alignment. Neurocomputing 2019b;325:211–24.

[btaf319-B8] Evans WE , McLeod HL. Pharmacogenomics—drug disposition, drug targets, and side effects. N Engl J Med 2003;348:538–49. 12571262 10.1056/NEJMra020526

[btaf319-B9] Galeano D , Li S, Gerstein M et al Predicting the frequencies of drug side effects. Nat Commun 2020;11:4575. 32917868 10.1038/s41467-020-18305-y PMC7486409

[btaf319-B10] Guo F , Guan R, Li Y et al Foundation models in bioinformatics. Natl Sci Rev 2025;12:nwaf028. page 40078374 10.1093/nsr/nwaf028 PMC11900445

[btaf319-B11] He K , Zhang X, Ren S et al Deep residual learning for image recognition. In *Proceedings of the IEEE conference on computer vision and pattern recognition*, 2016, 770–8.

[btaf319-B12] Jaeger S , Fulle S, Turk S. Mol2vec: unsupervised machine learning approach with chemical intuition. J Chem Inf Model 2018;58:27–35. 29268609 10.1021/acs.jcim.7b00616

[btaf319-B13] Kuhn M , von Mering C, Campillos M et al Stitch: interaction networks of chemicals and proteins. Nucleic Acids Res 2008;36:D684–8. 18084021 10.1093/nar/gkm795 PMC2238848

[btaf319-B14] Kuhn M , Campillos M, Letunic I et al A side effect resource to capture phenotypic effects of drugs. Mol Syst Biol 2010;6:343. 20087340 10.1038/msb.2009.98 PMC2824526

[btaf319-B15] Liang H , Chen L, Zhao X et al Prediction of drug side effects with a refined negative sample selection strategy. Comput Math Methods Med 2020;2020:1573543. 32454877 10.1155/2020/1573543 PMC7232712

[btaf319-B16] Liu W , Zhang J, Qiao G et al Hmmf: a hybrid multi-modal fusion framework for predicting drug side effect frequencies. BMC Bioinformatics 2024;25:196. 38769492 10.1186/s12859-024-05806-6 PMC11555943

[btaf319-B17] Lovrić M , Molero JM, Kern R. Pyspark and rdkit: moving towards big data in cheminformatics. Mol Inform 2019;38:e1800082. 30844132 10.1002/minf.201800082

[btaf319-B18] Marx V. Seeing data as t-sne and umap do. Nat Methods 2024;21:930–3. 38789649 10.1038/s41592-024-02301-x

[btaf319-B19] Masumshah R , Eslahchi C. Dpsp: a multimodal deep learning framework for polypharmacy side effects prediction. Bioinform Adv 2023;3:vbad110. 37701676 10.1093/bioadv/vbad110 PMC10493180

[btaf319-B20] Masumshah R , Aghdam R, Eslahchi C. A neural network-based method for polypharmacy side effects prediction. BMC Bioinformatics 2021;22:385. 34303360 10.1186/s12859-021-04298-y PMC8305591

[btaf319-B21] Oun R , Moussa YE, Wheate NJ. The side effects of platinum-based chemotherapy drugs: a review for chemists. Dalton Trans 2018;47:6645–6653. 29632935 10.1039/c8dt00838h

[btaf319-B22] Park S , Lee S, Pak M et al Dual representation learning for predicting drug-side effect frequency using protein target information. IEEE J Biomed Health Inform 2025;29:1817–27. 38241108 10.1109/JBHI.2024.3350083

[btaf319-B23] Tatonetti NP , Ye PP, Daneshjou R et al Data-driven prediction of drug effects and interactions. Sci Transl Med 2012;4:125ra31. 10.1126/scitranslmed.3003377 PMC3382018 22422992

[btaf319-B24] Wishart DS , Feunang YD, Guo AC et al Drugbank 5.0: a major update to the drugbank database for 2018. Nucleic Acids Res 2018;46:D1074–82. 29126136 10.1093/nar/gkx1037 PMC5753335

[btaf319-B25] Xu X , Yue L, Li B et al Dsgat: predicting frequencies of drug side effects by graph attention networks. Brief Bioinform 2022;23:bbab586. 35043189 10.1093/bib/bbab586

[btaf319-B26] Zhao H , Zheng K, Li Y et al A novel graph attention model for predicting frequencies of drug–side effects from multi-view data. Brief Bioinform 2021;22:bbab239. 34213525 10.1093/bib/bbab239

[btaf319-B27] Zhao H , Wang S, Zheng K et al A similarity-based deep learning approach for determining the frequencies of drug side effects. Brief Bioinform 2022;23:bbab449. 34718402 10.1093/bib/bbab449

[btaf319-B28] Zhao H , Zhong J, Liang X et al Application of machine learning in drug side effect prediction: databases, methods, and challenges. Front Comput Sci 2025;19:195902.

[btaf319-B29] Zhao X , Chen L, Lu J. A similarity-based method for prediction of drug side effects with heterogeneous information. Math Biosci 2018;306:136–44. 30296417 10.1016/j.mbs.2018.09.010

[btaf319-B30] Zhao X , Chen L, Guo Z-H et al Predicting drug side effects with compact integration of heterogeneous networks. CBIO 2019;14:709–20.

