# Supplementary material for: A deep learning-based method for predicting the frequency classes of drug side effects based on multi-source similarity fusion
Source: Bioinformatics. 2025 May 27;41(6):btaf319. doi: 10.1093/bioinformatics/btaf319 (PMC12141199; doi:10.1093/bioinformatics/btaf319)
Supplement: btaf319_Supplementary_Data [file btaf319_supplementary_data.pdf]

Supplementary Information of "A Deep Learning-based Method for Predicting the Frequency Classes of Drug Side Effects Based on Multi-Source Similarity Fusion"

1 Dataset

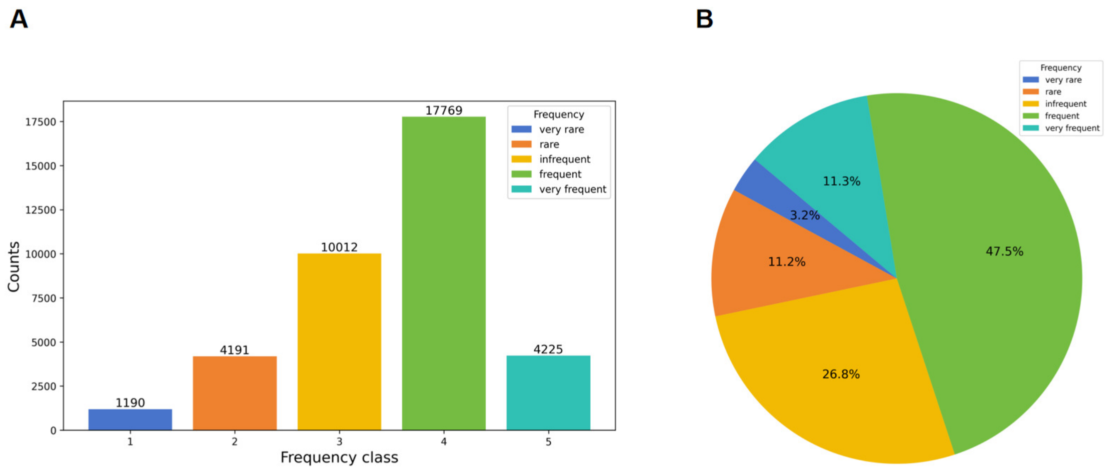

Supplementary Fig S1: The number and proportion of samples for each drug side effect frequency class in the benchmark dataset. (A) The distribution of samples across each frequency class. (B) The proportion of samples in each frequency class relative to the total number of samples.

2 Hyperparameter analysis

Supplementary Table S1: Effect of the learning rate on the predictive performance of MSSF evaluated via ten-fold cross-validation on the benchmark dataset.

Learning rate	ACC	Weighted F1	Macro F1	Kappa	MCC	Precision	Recall	AUPR
1e-3	0.6790	0.6716	0.6182	0.5113	0.5139	0.6515	0.6030	0.6674
5e-4	0.7121	0.7097	0.6716	0.5686	0.5695	0.6906	0.6589	0.7209
1e-4	**0.7246**	**0.7212**	**0.6809**	**0.5847**	**0.5863**	**0.7046**	**0.6641**	**0.7272**
5e-5	0.7228	0.7195	0.6795	0.5828	0.5841	0.6978	0.6677	0.7269
1e-5	0.7169	0.7104	0.6641	0.5684	0.5717	0.7012	0.6459	0.7229

Supplementary Table S2: The effect of the dropout rate on the predictive performance of MSSF evaluated via ten-fold cross-validation on the benchmark dataset.

dropout rate	ACC	Weighted F1	Macro F1	Kappa	Mcc	Precision	Recall	AUPR
0.1	0.7081	0.7043	0.6622	0.5599	0.5614	0.6834	0.6495	0.6987
0.2	0.7154	0.7110	0.6662	0.5692	0.5711	0.7005	0.6450	0.7091
0.3	0.7199	0.7162	0.6736	0.5771	0.5786	0.7050	0.6535	0.7191
0.4	**0.7246**	**0.7212**	**0.6809**	**0.5847**	**0.5863**	**0.7046**	**0.6641**	**0.7272**
0.5	0.7181	0.7152	0.6752	0.5766	0.5781	0.6958	0.6624	0.7250
0.6	0.7095	0.7081	0.6660	0.5670	0.5675	0.6759	0.6588	0.7112
0.7	0.6862	0.6794	0.6205	0.5209	0.5235	0.6769	0.5912	0.6694
0.8	0.6526	0.6241	0.5208	0.4486	0.4601	0.6317	0.4929	0.6124
0.9	0.5984	0.5256	0.3134	0.3312	0.3540	0.3516	0.3362	0.4992

Supplementary Table S3: The effect of the batch size on the predictive performance of MSSF evaluated via ten-fold cross-validation on the benchmark dataset.

batch size	ACC	Weighted F1	Macro F1	Kappa	MCC	Precision	Recall	AUPR
32	0.7166	0.7159	0.6794	0.5807	0.5810	0.6787	0.6828	0.7213
64	0.7230	0.7206	**0.6831**	0.5847	0.5857	0.6991	**0.6733**	**0.7273**
128	**0.7246**	**0.7212**	0.6809	**0.5847**	**0.5863**	**0.7046**	0.6641	0.7272
256	0.7193	0.7158	0.6744	0.5770	0.5786	0.7014	0.6574	0.7204

Supplementary Table S4: The effect of γ on the predictive performance of MSSF evaluated via ten-fold cross-validation on the benchmark dataset.

γ	ACC	Weighted F1	Macro F1	Kappa	MCC	Precision	Recall	AUPR
1e-1	0.6508	0.6282	0.4665	0.4559	0.4635	0.5038	0.4618	0.5859
1e-2	0.7110	0.7086	0.6674	0.5670	0.5681	0.6879	0.6550	0.7109
1e-3	0.7185	0.7154	0.6744	0.5772	0.5788	0.6899	0.6646	**0.7276**
1e-4	0.7202	0.7175	0.6778	0.5806	0.5819	0.6952	**0.6689**	0.7244
1e-5	**0.7246**	**0.7212**	**0.6809**	**0.5847**	**0.5863**	**0.7046**	0.6641	0.7272

Supplementary Table S5: The effect of d on the predictive performance of MSSF evaluated via ten-fold cross-validation on the benchmark dataset.

d	ACC	Weighted F1	Macro F1	Kappa	MCC	Precision	Recall	AUPR
16	0.7121	0.7080	0.6503	0.5661	0.5673	0.6835	0.6327	0.6997
32	0.7162	0.7124	0.6689	0.5721	0.5737	0.6881	0.6567	0.7170
64	**0.7246**	**0.7212**	**0.6809**	**0.5847**	**0.5863**	**0.7046**	0.6641	0.7272
128	0.7199	0.7162	0.6759	0.5778	0.5794	0.6937	**0.6650**	**0.7278**
256	0.7181	0.7144	0.6734	0.5757	0.5776	0.6961	0.6616	0.7233

Supplementary Table S6: The effect of the learning rate on the predictive performance evaluated on the independent test set.

Learning rate	ACC	Weighted F1	Macro F1	Kappa	MCC	Precision	Recall	AUPR
1e-3	0.5587	0.5085	0.3684	0.1999	0.2260	**0.5522**	0.3334	0.3775
5e-4	**0.5611**	0.4824	0.3526	0.1862	0.2291	0.5182	0.3448	**0.3920**
1e-4	0.5569	0.5079	0.3665	0.2050	0.2298	0.4728	0.3402	0.3842
5e-5	0.5606	**0.5282**	**0.3940**	**0.2425**	**0.2549**	0.4704	**0.3681**	0.3897
1e-5	0.5530	0.4866	0.3127	0.1770	0.2060	0.4834	0.2989	0.3574

Supplementary Table S7: The effect of the dropout rate on the predictive performance evaluated on the independent test set.

Dropout rate	ACC	Weighted F1	Macro F1	Kappa	MCC	Precision	Recall	AUPR
0.1	0.5592	0.4874	0.3332	0.1849	0.2194	0.4928	0.3220	0.3766
0.2	0.5606	**0.5282**	**0.3940**	**0.2425**	**0.2549**	0.4704	**0.3681**	**0.3897**
0.3	0.5562	0.4946	0.3716	0.1958	0.2281	**0.4941**	0.3560	0.3790
0.4	**0.5615**	0.4936	0.3339	0.1896	0.2253	0.4906	0.3169	0.3700
0.5	0.5521	0.4912	0.3508	0.1853	0.2138	0.4569	0.3339	0.3617
0.6	0.5533	0.4704	0.3182	0.1630	0.1998	0.4826	0.3059	**0.3855**
0.7	0.5471	0.4415	0.2541	0.1183	0.1706	0.3989	0.2639	0.3537
0.8	0.5363	0.4390	0.2098	0.1147	0.1471	0.2566	0.2381	0.3213
0.9	0.5341	0.4284	0.1965	0.0969	0.1329	0.1952	0.2297	0.3119

Supplementary Table S8: The effect of the batch size on the predictive performance evaluated on the independent test set.

Batch size	ACC	Weighted F1	Macro F1	Kappa	MCC	Precision	Recall	AUPR
32	0.5348	0.4960	0.3556	0.1938	0.2080	0.4018	0.3459	0.3555
64	**0.5606**	**0.5282**	**0.3940**	**0.2425**	**0.2549**	**0.4704**	**0.3681**	**0.3897**
128	0.5597	0.5031	0.3559	0.2091	0.2340	0.4667	0.3432	0.3656
256	0.5572	0.4724	0.2753	0.1563	0.2017	0.4927	0.2743	0.3712

Supplementary Table S9: The effect of γ on the predictive performance evaluated on the independent test set.

γ	ACC	Weighted F1	Macro F1	Kappa	MCC	Precision	Recall	AUPR
1e-1	0.5473	0.4551	0.2435	0.1370	0.1803	0.4083	0.2606	0.3064
1e-2	0.5493	0.4398	0.2836	0.1112	0.1796	0.5623	0.2846	0.3818
1e-3	0.5586	0.4804	0.3167	0.1656	0.2098	0.5128	0.3022	0.3894
1e-4	**0.5661**	0.4828	0.3214	0.1808	0.2268	**0.6289**	0.3078	**0.4005**
1e-5	0.5606	**0.5282**	**0.3940**	**0.2425**	**0.2549**	0.4704	**0.3681**	0.3897

Supplementary Table S10: The effect of d on the predictive performance evaluated on the independent test set.

d	ACC	Weighted F1	Macro F1	Kappa	MCC	Precision	Recall	AUPR
16	0.5575	0.4757	0.3159	0.1778	0.2135	**0.5203**	0.3140	0.3723
32	0.5606	**0.5282**	**0.3940**	**0.2425**	**0.2549**	0.4704	**0.3681**	**0.3897**
64	0.5547	0.4648	0.2997	0.1452	0.1979	0.4742	0.2953	0.3722
128	0.5567	0.4987	0.3613	0.1984	0.2263	0.4702	0.3515	0.3830
256	**0.5671**	0.4860	0.3106	0.1931	0.2319	0.4971	0.3097	0.3745

3 The calculation process of evaluation metrics

Accuracy (ACC) is calculated as follows:

$$\text{ACC} = \frac{N_{true}}{N} \tag{1}$$

where N_{true} represents the number of correctly predicted samples, and N is the total number of samples. Cohen's Kappa Score (κ) is calculated as:

$$\kappa = \frac{p_o - p_e}{1 - p_e} \tag{2}$$

where the model's prediction scores and the true labels of samples are considered as two raters, p_o represents the actual observed agreement between the two raters, while p_e represents their expected agreement [1]. The weighted F1 score (Weighted F1), macro F1 score (Macro F1), macro precision (Precision) and macro recall (Recall) can be calculated as follows:

$$P_i = \frac{TP_i}{TP_i + FP_i}, R_i = \frac{TP_i}{TP_i + FN_i} \tag{3}$$

$$\text{Weighted F1} = \sum_{i=1}^{5}(\frac{2 \cdot P_i \cdot R_i}{P_i + R_i})\frac{N_i}{N} \tag{4}$$

$$\text{Macro F1} = \frac{1}{5}\sum_{i=1}^{5}(\frac{2 \cdot P_i \cdot R_i}{P_i + R_i}) \tag{5}$$

$$\text{Precision} = \frac{1}{5}\sum_{i=1}^{5} P_i \tag{6}$$

$$\text{Recall} = \frac{1}{5}\sum_{i=1}^{5} R_i \tag{7}$$

where TP_i, FP_i, TN_i, and FN_i represent the number of true positive samples, false positive samples, true negative samples, and false negative samples in class i, respectively. N_i represents the number of samples in class i, and N is the total number of samples. Similarly, the macro AUPR (AUPR) is computed as the arithmetic mean of the per-class AUPR values, where each class's AUPR is the area under its own precision–recall curve. Matthews Correlation Coefficient (MCC) is calculated as:

$$Mcc = \frac{c \cdot s - \sum_{i=1}^{5}(p_i \cdot t_i)}{\sqrt{(s^2 - \sum_{i=1}^{5} p_i^2)(s^2 - \sum_{i=1}^{5} t_i^2)}} \tag{8}$$

where c is the total number of correctly predicted samples, s is the total number of samples, p_i represents the number of samples predicted for class i, and t_i represents the number of samples in class i.

4 Kernel density estimation and maximum likelihood estimation

We employ Kernel Density Estimation (KDE) to estimate the Probability Density Function (PDF) of the prediction scores of the samples for each class and assign the prediction scores of the regression models to the predefined class based on the maximum likelihood principle. For class i, there is a set of predicted scores $\{x_k\}_{k=1}^{n_i}$, where x_k is the prediction score of the k-th sample, and n_i is the number of samples in class i. The PDF of the prediction score x for class i can be estimated using KDE:

$$P_i(x) = \hat{f}_i(x) = \frac{1}{n_i h}\sum_{k=1}^{n_i} K\left(\frac{x - x_k}{h}\right)$$

where h is the bandwidth parameter, and $K(\cdot)$ is a Gaussian kernel function which can be defined as:

$$K(u) = \frac{1}{\sqrt{2\pi}} e^{-\frac{u^2}{2}}$$

The classification decision is made by maximizing the likelihood, assigning the sample with prediction score x to the class $\hat{i}$ that maximizes the estimated probability density functions:

$$\hat{i} = \arg\max_i P_i(x)$$

5 Case study experiment results

Supplementary Table S11: Top 10 potential side effects of haloperidol.

Side effect	Evidence
erythema nodosum	NA
acute myocardial infarction	OFFSIDERS
prurigo	OFFSIDERS
pneumothorax	OFFSIDERS
foetor hepaticus	NA
hypertriglyceridaemia	OFFSIDERS
nephropathy toxic	OFFSIDERS
atelectasis	OFFSIDERS
anuria	OFFSIDERS
crohn's disease	OFFSIDERS

Supplementary Table S12: Top 10 potential side effects of trimethoprim.

Side effect	Evidence
osteonecrosis	OFFSIDERS
depressed level of consciousness	OFFSIDERS
cardiogenic shock	OFFSIDERS
hepatotoxicity	OFFSIDERS
fracture	OFFSIDERS
alveolitis allergic	OFFSIDERS
polyneuropathy	OFFSIDERS
retroperitoneal haemorrhage	OFFSIDERS
aneurysm	OFFSIDERS
nephritis	OFFSIDERS

Supplementary Table S13: Top 10 potential side effects of amoxicillin.

Side effect	Evidence
cardiogenic shock	OFFSIDERS
liver disorder	OFFSIDERS
lung infiltration	OFFSIDERS
pleuritic pain	OFFSIDERS
hepatocellular injury	OFFSIDERS
aplastic anaemia	OFFSIDERS
laryngeal oedema	OFFSIDERS
alveolitis allergic	OFFSIDERS
nuchal rigidity	OFFSIDERS
gastrointestinal perforation	OFFSIDERS

References

[1] Artstein, R., & Poesio, M. (2008). Inter-coder agreement for computational linguistics. Computational linguistics, 34(4), 555-596.
